# Necrology

**Published:** 1902-02

**Authors:** 


### NECROLOGY.

### ROBERT JACKSON SAUNDERS, V.S.

ON January 22, 1902, at Highland Station, West Roxbury, Mass., Dr. R. J. Saunders, died from heart lesions as a complication of rheumatism, aged sixty-six years. He was one of the oldest members of the A. V. M. A., and though a self-made man, was a thoroughly conscientious and studious practitioner, and gained the respect and good-will of all who knew him. When the A. V. M A. held its annual meetings in Boston, and the semi-annual ones in New York in its early history, it was rare that Dr. Saunders was ever absent, and he was a frequent contributor to the programme. He was a brother to the late William

Saunders, the father of Frederick and an uncle of the late John S. Saunders. He gave up active practice about two years ago on account of rheumatism, but was only confined to the house for three days. Several others of the same family have entered the veterinary profession and stood for much of the profession's advancement in the "Bay State."

---

### WILLIAM H. PROPHETT, D.V.S.

The above-named veterinarian died at Suffield, Conn., January 17, 1902. His death followed a long period of illness, which had forced his retirement from active practice some five years before. The deceased was a graduate of the American Veterinary College, class of 1885, and had practised at Bridgeport, Conn., for the greater part of his professional career. His son, James H. Prophett, veterinarian, a graduate of the same college as his father, succeeds to his business.

---

### GEORGE S. CROSSMAN, V.S.

At Malone, N. Y., December 29, 1902, Dr. George S. Crossman died after an illness of about two years, at the age of forty-one years. He was a student in the agricultural course at Cornell University, but gained his veterinary degree at the McGill University, Veterinary Department. Dr. Crossman was known as a skilful and successful practitioner, and was keenly interested in the advancement of his profession. He was an active and potential factor in Democratic politics in the Empire State. He was buried with Masonic ceremonies and with similar rites by the Odd Fellows, with both of which Orders he was actively identified.

---

At St. Thomas, Ontario, January 13, 1902, Dr. A. H. King, graduate of the Ontario Veterinary College, class of 1886.

---

### CIVIL SERVICE EXAMINATIONS.

Examinations for meat-inspectors will be held in all the large cities of the United States on February 27, 1902, by the United States Civil Service Commission. All applicants must be twenty years old or over and be graduates of veterinary colleges.

